# Human CD64-targeted non-viral siRNA delivery system for blood monocyte gene modulation

**DOI:** 10.1038/srep42171

**Published:** 2017-02-07

**Authors:** Seok-Beom Yong, Hyung Jin Kim, Jang Kyoung Kim, Jee Young Chung, Yong-Hee Kim

**Affiliations:** 1 Department of Bioengineering, Institute for Bioengineering and Biopharmaceutical Research, BK 21 Plus Future Biopharmaceutical Human Resources Training and Research Team, Hanyang University, 133-791 Seoul, Republic of Korea

## Abstract

A subset of phagocytes including inflammatory monocytes in blood migrate and give rise to macrophages in inflammatory tissues which generated the idea that blood monocytes are the therapeutic targets for drug delivery. Fc gamma receptor I (CD64) is a membrane receptor for the Fc region of immunoglobulin G, primarily expressed on monocyte-lineage, and H22 a monoclonal antibody for human CD64 had shown rapid blood monocyte binding and occupation in clinical studies. Small interfering RNA-mediated gene silencing as a therapeutic has been proposed and is a promising strategy in terms of its “knock-down” ability on the target gene prior to translation. However, its instability and off-targeting effect must be overcome for success in clinical studies. In this study, we developed a non-viral delivery system composed of oligo-nona-arginine (9R) and anti-human CD64 single chain antibodies (H22) for human monocyte-specific siRNA delivery. A targeted and efficient siRNA delivery mediated by anti-CD64 scFv-9R was observed in CD64 positive human leukemia cells, THP-1. With primary human blood cells, anti-CD64 scFv-9R mediated gene silencing was quantitatively confirmed representing blood monocyte selective gene delivery. These results demonstrate the potential of anti-CD64 scFv-9R mediated siRNA delivery for the treatment of human inflammatory diseases via blood monocytes gene delivery.

Mononuclear phagocytes are primary components of the immune system, and are involved with body homeostasis and immunological effector functions such as red blood cell clearance, cytokine release and foreign phagocytosis. Mononuclear phagocytes are considered a hindrance to drug delivery systems, as the actions of phagocyte often limit drug efficacy, mainly kupffer cells in the liver sequester and internalize administered nanoparticles and remove^1^. However, a subset of blood monocytes migrate to and accumulate in diseased areas and inflammatory tissues. This observations inspired another perspective, one in which the blood monocytes could be used as therapeutic targets for drug delivery. Inflammatory-monocytes, including Ly6c-high monocytes in mice and CD14-high monocytes in human are the main subsets known for CCR2-mediated accumulation in sites of inflammation such as infarcted cardiac^2^, adipose tissues^3^, brain myelitis^4^ and tumors^5^. Some papers have reported that cancer cell metastasis is also supported by inflammatory-monocytes^6^. Tissue accumulated monocytes can ultimately differentiate into inflammatory-macrophages to activate or modulate the inflammation. Consistent with this observation, siRNA-mediated chemokine receptor (CCR2) reduction in blood monocytes reduced tissue monocyte and macrophage accumulation resulting in lower levels of tissue damage and reduced-lesion sizes in mice disease models^7^. Recently even the tumor targeting effect of carbon nanotubes has been shown to be partially mediated and enhanced by inflammatory-monocytes^8^, consequently suggesting the therapeutic potential of blood monocytes-targeted and -mediated drug delivery for human diseases.

Small interfering RNAs are short, 21–24 nucleotide RNA fragments that regulate gene expression, primarily in the cytosol. Compared with antibodies and synthetic drugs, siRNAs are capable of “knocking down” target genes with high specificity before translation^9^. Like other drugs siRNAs require a delivery system for improved efficacy and reduced side effects *in vivo*. Over the past few decades targeted siRNA delivery systems have been developed depending on targeting moieties such as peptides^10^, antibodies^11^, natural molecules^12^ and aptamers^13^. Single chain antibody (scFv), the antigen binding portion of antibody retains its high antigen binding affinity as whole antibody with smaller size and have been applied for targeted siRNA delivery. In papers of anti-CD7 scFv^14^ and anti-HBV Ag scFv^15^, anti-HIV glycoprotein scFv^11^ demonstrated *in vivo* target specificity of scFv-mediated siRNA delivery. Recently, Her2-targeted scFv-protamine fusion protein-mediated siRNA delivery suppressed growth and metastasis of human primary breast cancer in mice^16^.

The Fc gamma receptor family encompasses membrane glycoprotein receptor for the Fc region of immunoglobulin G and distributed on diverse type of cells, from immune cells to endothelial, epithelial cells. Human Fc gamma receptor I (CD64) expression is primarily on monocytes and cells of macrophage lineages^17^,^18^. The H22, a monoclonal antibody targeting human CD64 have been applied for immunotoxins ablating CD64-positive monocytic leukemia^19^, and phase I clinical studies showed rapid monocyte binding of H22 in circulation^20^,^21^.

In this study, we investigate CD64-targeted siRNA delivery for human blood monocytes gene silencing. We conjugated nucleic acid binding nona-arginine peptide with an anti-human CD64 single chain antibody (H22). Anti-CD64 scFv-9R showed target-receptor mediated siRNA delivery and gene silencing in a human monocytic cell line (THP-1). In human peripheral blood cells (PBMCs), anti-CD64 scFv-9R showed monocytes-selective uptake and gene silencing without significant toxicity *ex vivo*. With a human monocyte engrafted mouse, scFv-9R/siRNA complex demonstrated human monocyte-targeted delivery *in vivo*, and collectively demonstrated the potential of CD64 targeted scFv-9R for blood monocyte siRNA delivery in human inflammatory disease.

## Results

### Schematic illustration of CD64-targeted siRNA delivery system

**Figure 1:**
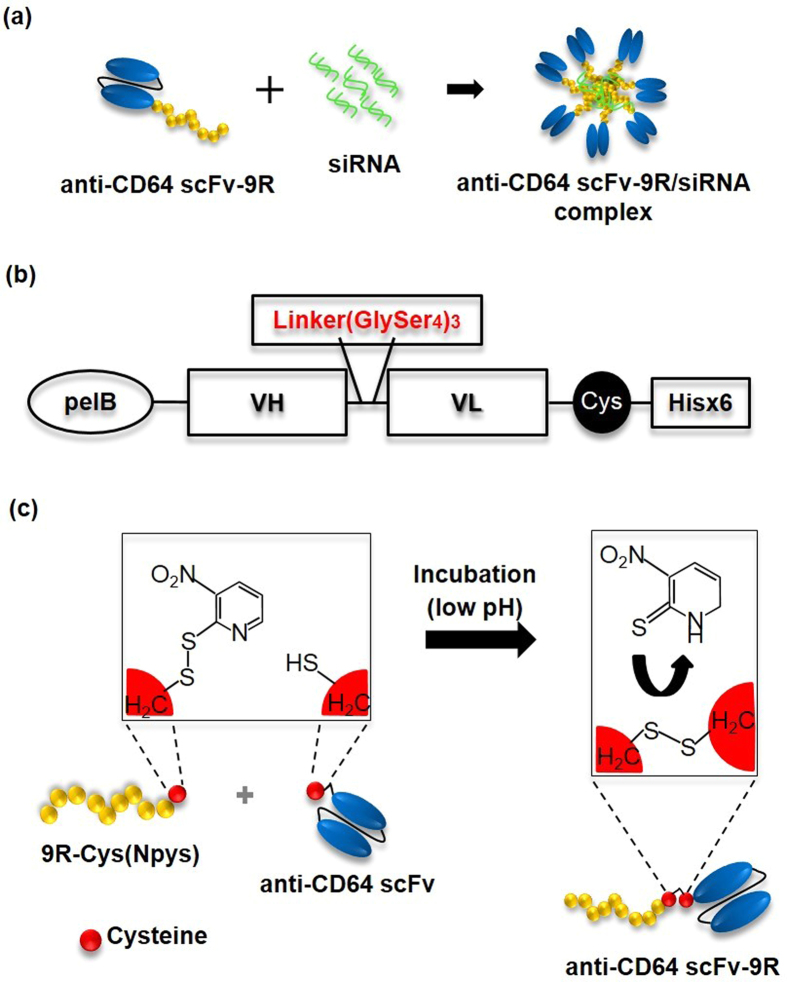
Schematic illustration and vector map of CD64-targeted siRNA delivery system. (**a**) CD64-targeted siRNA delivery system. (**b**) Anti-CD64 scFv structure. (**c**) Chemistry of oligo-D-arginine conjugation to anti-CD64 scFv.

It has been reported that the monoclonal antibody for human CD64, H22 binds to different epitopes from IgG binding, representing target binding in the presence of serum IgG^22^, and H22 has shown rapid blood monocyte binding in phase I clinical trials^20^,^21^. Additionally toxins conjugated with H22 were efficiently internalized into CD64 positive leukemia cells and induced cancer cell apoptosis^23^. Based on these studies, we developed a CD64-targeted non-viral carrier consisting of a single chain antibody of H22 (anti-CD64 scFv) and 9-mer oligo-D-arginine (9R) as shown in Fig. 1a, having triple benefits of target specificity, gene stabilization and cell penetrating efficiency. For construction of the anti-CD64 scFv-expressing vector, the gene sequence encoding anti-CD64 scFv was cloned into pET21a. Anti-CD64 scFv contains a signal peptide (pelB) for periplasmic localization, which facilitates protein folding, linker sequence (GlySer_4_)_3_ and cysteine to create a disulfide bond with the terminal cysteine of oligo-nona-D-arginine (Fig. 1b). Oligo-arginine has been widely used for protein^24^,^25^ and gene delivery^10^,^26^,^27^. In particular, D-form oligo-arginine has been reported to be more effective in improving gene transfection efficiency and *in vivo* stability than L-form. Reducible polymers based on the D-form oligo-arginine have shown significantly improved transfection efficiency compared with PEI and lipofectamine^28^. As described in reference^14^, oligo-D-arginine with an (Npys) cysteine residue was incubated with anti-CD64 scFv in a low pH phosphate buffer for conjugation and dialyzed to remove unconjugated oligo-arginine (MWCO; 12-14,000 Da) (Fig. 1c).

### Characterization and functionality of purified anti-CD64 scFv and anti-CD64 scFv-9R conjugate

**Figure 2:**
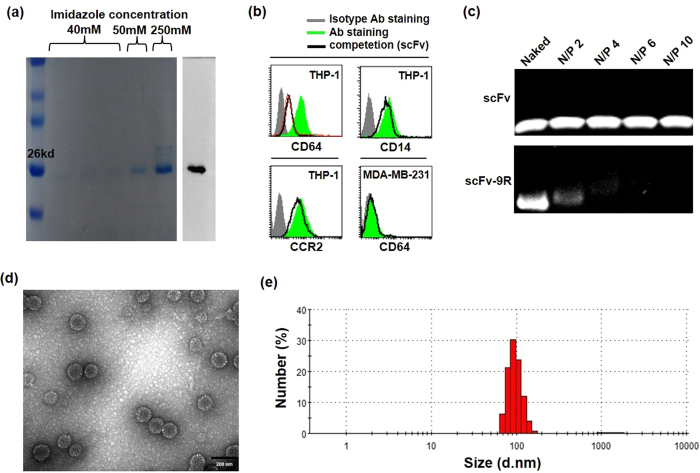
Characterization of purified anti-CD64 scFv and anti-CD64 scFv-9R/siRNA. (**a**) Purified anti-CD64 scFv was identified at the position of 26–27 kDa by SDS-PAGE and western blot analysis. (**b**) Competition of purified anti-CD64 scFv with commercial CD64 antibody (FITC) for THP-1 cell binding. Pretreatment with 2 μg anti-CD64 scFv reduced commercial CD64 antibody (FITC) binding in THP-1 cells (CD64 positive), while pretreatment with the same amount of anti-CD64 scFv did not reduce binding of other antibodies (CD14 antibody (PE), CCR2 antibody (PE)) in THP-1 cells. MDA-MB-231 cell (CD64 negative) demonstrated no binding and competition with anti-CD64 scFv. (**c**) Gene retardation efficiency of anti-CD64 scFv-9R. The siLuci (40 pmol) were mixed with anti-CD64 scFv-9R and anti-CD64 scFv at various N/P ratios at room temperature for 15 min and loaded on 2% agarose gel. (**d**) TEM image of anti-CD64 scFv-9R/siRNA. The siLuci (150 pmol) were mixed with anti-CD64 scFv-9R (NP ratio 4) and analyzed with transmission electron microscopy. (**e**) Nano-size distribution of anti-CD64 scFv-9R/siRNA (siLuci = 150 pmol, N/P ratio 4).

The anti-CD64 scFv expressed in a bacterial system (BL21 (DE3)) and purified using affinity chromatography provided a single band around 26 kDa in SDS-PAGE. The western blot result stained with an anti-histidine tag antibody showed the same band location as SDS-PAGE (Fig. 2a). After oligo arginine conjugation, anti-CD64 scFv-9R conjugate showed a single band at the expected position in SDS-PAGE and The conjugation efficiency was ~77% as quantified using a free thiol quantification kit (Fig. S1b). The functionality of anti-CD64 scFv was tested by measuring competitive binding with a commercial human CD64 antibody in CD64 positive THP-1 and CD64 negative MDA-MB-231 cells. As shown in Fig. 2b, THP-1 cells pre-incubated with anti-CD64 scFv (black thick line) demonstrated reduced commercial CD64 antibody (FITC) binding, representing that anti-CD64 scFv competes with commercial CD64 antibody. On the other hand, no competitive binding was observed between anti-CD64 scFv and other antibodies against CD14 and CCR2 in THP-1 cells. CD64 negative MDA-MB-231 cells did not show binding affinities for anti-CD64 scFv and commercial CD64 antibody. In the siRNA retardation study, anti-CD64 scFv-9R conjugate retarded the siRNA in the N/P ratio above 4 in contrast to anti-CD64 scFv (Fig. 2c). The size of anti-CD64 scFv-9R/siRNA was analyzed with transmission electron microscopy (Fig. 2d) and DLS nano-size distribution (Fig. 2e).

### Anti-CD64 scFv-9R mediated siRNA delivery into THP-1

**Figure 3:**
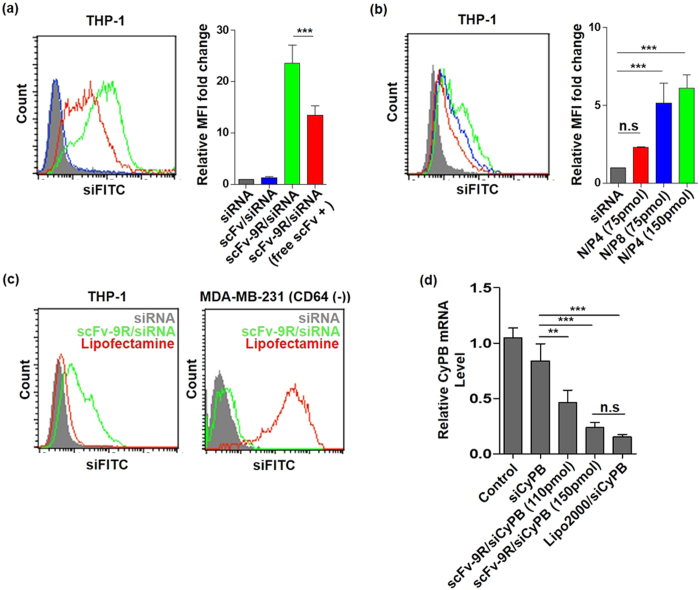
Anti-CD64 scFv-9R mediated siRNA delivery into THP-1. (**a**) Anti-CD64 scFv-9R mediated cell binding of siRNA. The 75 pmol siLuci (FITC) complexed with anti-CD64 scFv-9R in N/P ratio 4 were bound and internalized into THP-1 via CD64 after 1 hour of incubation and blocked in presence of free anti-CD64 scFv. Same amount of siLuci (FITC) complexed with anti-CD64 scFv did not bind and internalized into THP-1. (**b**) Anti-CD64 scFv-9R mediated cellular internalization of siLuci (FITC) is in a concentration dependent manner (13 hours, N/P ratio = 4 or 8, siLuci = 75–150 pmol). (**c**) Target receptor mediated siRNA delivery of anti-CD64 scFv-9R. The 150 pmol siLuci (FITC) complexed with anti-CD64 scFv-9R in N/P ratio 4 delivered to THP-1, not for MDA-MB-231 (CD64 (−)). (**d**) Gene silencing effect of anti-CD64 scFv-9R mediated siRNA delivery. The siCypB (110 pmol and 150 pmol) were complexed with anti-CD64 scFv-9R in N/P ratio 4 and treated, mRNA levels were measured using RT-PCR (cyclophilin B mRNA level was normalized by beta-actin mRNA). Each graph represents one of three independent experiments. All data are presented as the mean ± standard deviation (n = 3, **p < 0.01, ***p < 0.001 by One-way ANOVA and Tukey posttest).

To examine anti-CD64 scFv-9R mediated siRNA delivery in CD64 positive cells, we incubated the anti-CD64 scFv-9R/siRNA complex with human monocytic leukemia cell THP-1. After an incubation period of 30 min–1 hour, siRNA complexed with anti-CD64 scFv-9R bound or internalized in THP-1 cells with ~25 mean fluorescence intensity (MFI) fold change than that of naked siRNA or anti-CD64 scFv complexed siRNA. In the presence of free anti-CD64 scFv, anti-CD64 scFv-9R/siRNA complex showed decreased fluorescence intensity, means ‘target receptor-mediated’ and ‘scFv mediated’ cell association (Fig. 3a). In a longer time period (13 hours), anti-CD64 scFv-9R increased the cellular uptake of siRNA, with a mean fluorescence intensity 2 to 5 times greater than that of naked siRNA depending on the amount of anti-CD64 scFv-9R and siRNA (Fig. 3b). To confirm its CD64-target specificity, THP-1 and MDA-MB-231 (CD64 negative) were treated with anti-CD64 scFv-9R/siRNA complex. THP-1 cells internalized anti-CD64 scFv-9R/siRNA though lipofectamine2000/siRNA showed lower internalization. However MDA-MB-231 did not internalize anti-CD64 scFv-9R/siRNA despite the high uptake of lipofectamine2000/siRNA (Fig. 3c). In addition, CHO-K1 cells (hamster ovary cells) were transfected with human CD64-expressing vector (CMV-promoter, origene) and treated with scFv-9R/siRNA complex. Compared with wild type (WT) CHO-K1 cells, transfected CHO-K1 cells demonstrated scFv-9R/siRNA (FITC) binding which means human CD64-mediated cell binding of scFv-9R. To confirm gene silencing effect of anti-CD64 scFv-9R mediated siRNA delivery, we employed cyclophilin B siRNA and analyzed with RT-PCR. As shown in Fig. 3d, anti-CD64 scFv-9R/siCypB complex reduced cyclophilin B mRNA in THP-1 cells comparable with lipofectamine/siCypB.

### Blood monocyte specific uptake and gene silencing of anti-CD64 scFv-9R/siRNA in human peripheral blood mononuclear cells

**Figure 4:**
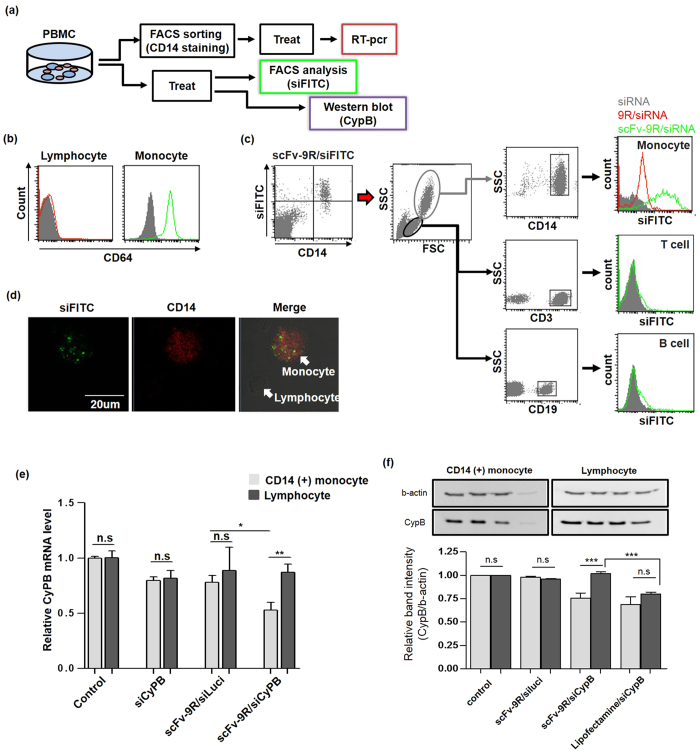
Blood monocyte selective gene silencing of the anti-CD64 scFv-9R/siRNA complex in human blood cell (PBMC). (**a**) Experimental scheme for PBMCs. (**b**) CD14 (+) monocyte specific CD64 expression in PBMC (CD14 antibody (FITC), CD64 antibody (PerCP-Cy5.5)). (**c**) Anti-CD64 scFv-9R/siLuci (FITC) complexes were mostly taken up by CD14 (+) monocytes (siLuci = 150 pmol, N/P ratio 5), oligo-D-arginine (siLuci = 150 pmol, N/P ratio 10). In detail, anti-CD64 scFv-9R/siLuci (FITC) complexes were not internalized by lymphocytes (SSC gated) including CD3 (+) T cells and CD19 (+) B cells in PBMCs. Same amount of siLuci (FITC) complexed with oligo-D-arginine demonstrated lower, few monocytes internalization. (Graphs represent one of three independent experiments). (**d**) Confocal microscopy image of CD14 monocytes-internalized anti-CD64 scFv-9R/siRNA. Total PBMCs treated with anti-CD64 scFv-9R/siRNA (siFITC = 150 pmol, NP ratio 5) were stained with CD14 antibody and analyzed with confocal microscopy (CD14 antibody (PE), FITC-siRNA (FITC). (**e**) Human monocytes specific gene silencing effect of anti-CD64 scFv-9R (RT-PCR). The FACS sorted CD14 (+) monocytes and lymphocytes were treated with siCypB (110 pmol) complexed with anti-CD64 scFv-9R in N/P ratio 4 (cyclophilin B mRNA was normalized by beta-actin mRNA; data represented as the mean ± standard deviations, n = 3, **p < 0.01 by One-way ANOVA and Tukey posttest). (**f**) Anti-CD64 scFv-9R mediated the gene silencing effect in cyclophilin B protein. The siCypB (110 pmol) complexed with anti-CD64 scFv-9R in N/P ratio 4 reduced cyclophilin B expression in CD14 (+) monocytes to ~73% of control and anti-CD64 scFv-9R/siLuci group (Graphs represent the relative band intensity of cyclophilin B/beta-actin, compared to control group, n = 3, data represented as the mean ± standard deviation, ***p < 0.001 by One-way ANOVA and Tukey posttest).

In human blood, monocytes are known to express CD64, and human monocytes can be classified based on surface CD14 and CD16 expression. Human CD14-high monocytes are inflammatory-monocytes complement to Ly6c-high monocytes in mice^29^, accumulate in inflammatory sites migrated from spleen^2^ or bone marrow. To confirm monocyte specific siRNA delivery, we used human peripheral blood mononuclear cells (PBMC). In PBMCs, CD14 monocytes expressed CD64 on its surface, but not on the others (Fig. 4b). To test blood monocyte-targeted siRNA delivery, we incubated PBMCs with the anti-CD64 scFv-9R/siRNA complex. After 11–12 hours (4 hours of treat and additional 7–8 hours), the CD14 positive cell population took up anti-CD64 scFv-9R/siRNA complex and CD14 negative cell population did not. For further analysis, treated PBMCs were gated for cell lineage markers (CD14, CD3 and CD19). As shown in Fig. 4c, most of CD14 (+) cells took up anti-CD64 scFv-9R/siRNA complex, and very few CD3 (+) T cells and CD19 (+) B cells did. Total lymphocytes also did not take up anti-CD64 scFv-9R/siRNA complex. Consistent with FACS results, CD14 (+) cells took up anti-CD64 scFv 9R/siRNA complex in confocal microscopy imaging (Fig. 4d, Fig. S4). To examine gene silencing effects, CD14 and SSC based FACS sorting carried out (Fig. S5). Sorted CD14 (+) monocytes and lymphocytes were treated with the anti-CD64 scFv-9R/siCypB complex, and cyclophilin B mRNA was measured using RT-PCR. In comparison with lymphocytes, cyclophilin B mRNA was reduced in CD14 (+) cells only (~50% to control group), consistent with FACS analysis (Fig. 4e, **p < 0.01). In contrast, other groups showed similar cyclophilin B mRNA levels between CD14 (+) cells and lymphocytes (~80 to 90% to control groups). To measure gene silencing effect in protein level, cyclophilin B protein level was identified using western blots. As shown in Fig. 4f, scFv-9R/siCypB reduced cyclophilin B protein in CD14 (+) monocytes specifically. In contrast, Lipofectamine2000/siCypB complex reduced cyclophiln B protein both in lymphocytes and CD14 (+) monocytes. The siRNA for human CD45 (protein tyrosine phosphatase) was used^7^ for surface protein level silencing test and CD14 and SSC based gating strategy confirmed that CD14 (+) monocytes specific reduction in surface CD45 (*p < 0.05, mean fluorescence intensity value was ~80% compared to naked siCD45 group and anti-CD64 scFv-9R/siLuci group), but lymphocytes did not exhibit even a slight decrease in surface CD45 expression (Fig. S3).

### Toxicity of anti-CD64 scFv-9R/siRNA complex on human PBMC

**Figure 5:**
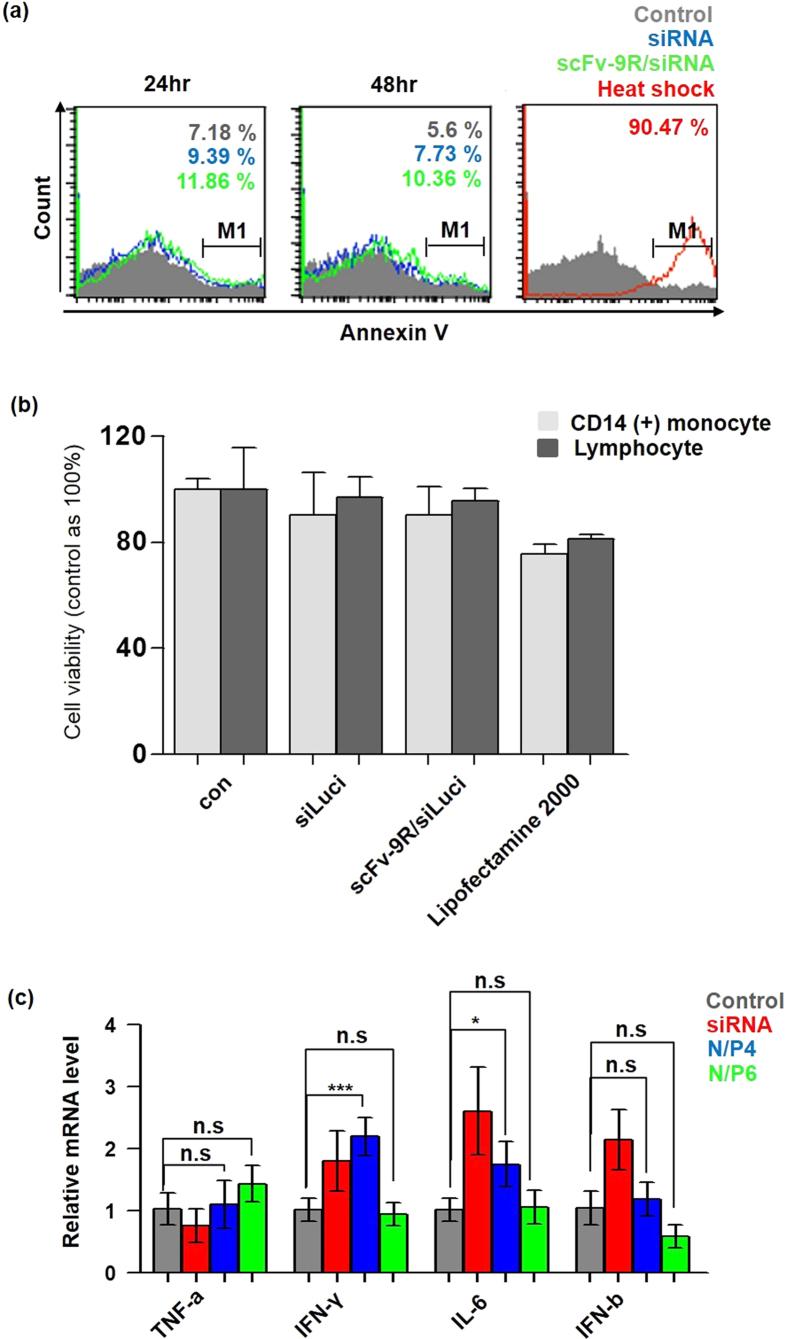
Toxicity of anti-CD64 scFv-9R/siRNA complex on PBMCs. (**a**) Annexin V staining for toxicity test. PBMCs were treated with siLuci (150 pmol) complexed with anti-CD64 scFv-9R in N/P ratio 4 and stained with Annexin V (PE) after 24 hours and 48 hours. Heat-shocked PBMCs were used as positive control. The numbers in each histogram indicates percentages of M1 gated cells. (**b**) MTT assay for toxicity test. CD14 (+) monocytes and lymphocytes were treated with siLuci (150 pmol) complexed with anti-CD64 scFv-9R in N/P ratio 4 and MTT assay was performed (Data presented as mean ± s.d., n = 3). (**c**) Immune response of PBMCs after 24 hours of treatment with the anti-CD64 scFv-9R/siRNA complex (siLuci = 150 pmol, data presented as the mean ± standard deviation, n = 3, n.s. = not significant by One-way ANOVA and Tukey posttest).

To test for cytotoxicity, anti-CD64 scFv-9R/siRNA complex treated PBMCs were stained with Annexin V (PE) and analyzed. In comparison with non-treated groups, and only siRNA treated groups, the anti-CD64 scFv-9R/siRNA complex treated group did not exhibit a significant change in Annexin V staining, indicating that anti-CD64 scFv-9R doesn’t have significant toxicity. As a positive control, heat-shocked PBMCs showed substantial change in Annexin V staining (Fig. 5a). Additional cell viability test was performed with MTT assay. As shown in Fig. 5b, sorted CD14 (+) monocytes and lymphocytes were treated with scFv-9R/siRNA complex in the same concentration as the knock-down experiments. Compared with other groups, scFv-9R/siRNA complex showed no significant toxicity. To examine immune response, inflammatory gene mRNA levels were measured^30^,^31^. The naked siRNA treated groups showed slight TNF-alpha down regulation and IFN-gamma, IFN-beta, IL-6 up regulation, thought to be the TLR signaling mediated response as previously reported^32^. The scFv-9R complexed siRNAs compromised the siRNA mediated inflammatory gene induction and is due to the sequestering of the scFv-9R complexed siRNAs inside in higher N/P ratio (Fig. 5c). These results indicate that the anti-CD64 scFv-9R/siRNA complex has mild toxicity in the indicated amount, and masking the siRNA’s natural immune response.

### *In vivo* human monocyte targeting effect of anti-CD64 scFv-9R/siRNA complex

**Figure 6:**
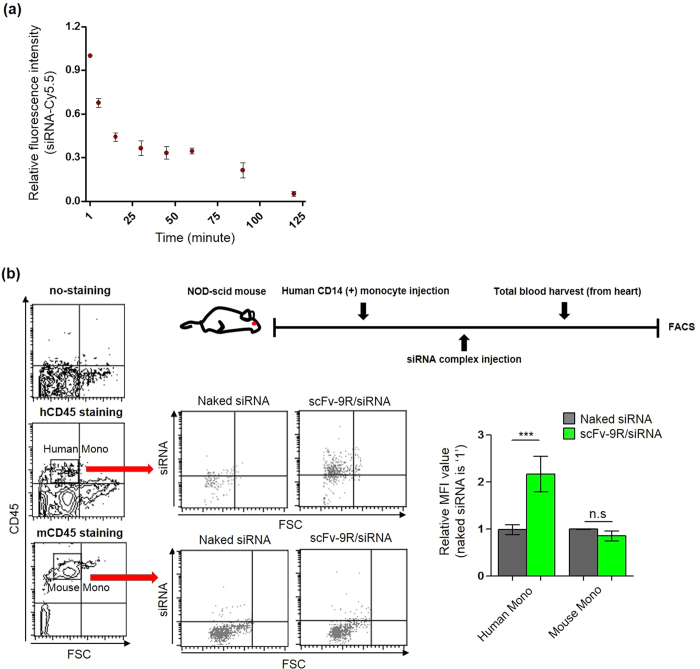
*In vivo* human monocyte targeting effect of anti-CD64 scFv-9R/siRNA complex. (**a**) *In vivo* blood profile (siRNA (Cy5.5) = 500 pmol, N/P ratio 4, Cy5.5 intensity at initial time point (30 sec–1 min) was indicated as 100%, data presented as the mean ± standard deviation, n = 3). (**b**) Flowcytometric analysis showed human monocyte (human CD45 positive cells) specific internalization of scFv-9R/siRNA complex in mouse blood cells with 2–3 times higher FITC fluorescence intensity compared with naked siRNA group. Mouse blood monocytes (mouse CD45 positive cells) showed no uptake of scFv-9R/siRNA complex (siRNA (FITC) = 500 pmol, N/P ratio 4, data presented as the mean ± standard deviation, by One-way ANOVA and Tukey posttest, n = 3).

To test *in vivo* targeting effect of scFv-9R/siRNA complex on human monocytes, human CD14 (+) monocyte-engrafted NOD-scid mice (5–7.5 × 10^5^ cells per mouse) were injected with scFv-9R/siRNA complex intravenously and after 1–2 hours, total blood cells were analyzed for siRNA uptake. As shown in Fig. 6b, the complex of scFv-9R with siRNA was taken-up by human monocytes (human CD45 staining). In contrast, mouse monocytes (mouse CD45 staining, FSC) showed no-uptake. As shown in Fig. 6a, scFv-9R/siRNA complex was cleared from blood after 2 hours^33^,^34^ (Fig. 6a).

## Discussion

Many papers proved that subsets of monocytes are the source of disease accumulating macrophages^2^,^3^,^4^,^5^, suggesting that blood circulating monocytes are appropriate targets for disease-targeted drug delivery. Phagocyte-targeted drug delivery, especially for monocyte and macrophage lineage have demonstrated its therapeutic effect in animal models^7^,^30^, and potential human clinical application. In our previous work, polymerization of D-form oligo-arginine with terminal cysteine residues (rPOA) improved its gene transfection ability for both siRNA & plasmid DNA. Despite high transfection efficiency, its non-target specificity limits local *in vivo* applications^35^,^36^. With the theory of human blood monocytes as drug delivery targets, we developed a non-viral siRNA delivery system based on a single chain antibody and oligo-arginine. Target receptor human CD64, a monocyte lineage-specific expression improves its target specificity. Some papers reported CD64 expression is elevated on circulating monocyte from patient with inflammatory disease and its expression is IFN-gamma signaling mediated^37^,^38^ resulting in inducible inflammatory conditions, and also IFN-gamma is known for its role in M1 macrophage polarization. For the goal of targeting, we employed H22 (scFv), anti-human CD64 antibody as a targeting moiety. H22 is different from IgG for Fc receptor binding in terms of binding epitope and Fc gamma receptor I (CD64) specificity rather than other type of Fc receptors (CD32, CD16)^22^, and this has been applied for the development of immunotoxins targeting CD64-positive human myeloid leukemia in previous studies^19^,^23^,^39^,^40^. In this study, anti-CD64 scFv-9R/siRNA complexes were characterized for their target receptor-mediated cell binding and uptake, and lipofectamine comparable gene silencing efficacy in THP-1. Experiments with human PBMCs demonstrates blood monocyte specific uptake and gene silencing *ex vivo* and human monocyte-targeting effect *in vivo*. However we found mild silencing effect on protein levels which may be attributed to the non-proliferative or quiescent state of monocyte *ex vivo* and limited endosome escape of oligo-arginine. Generally for non-proliferative primary cell gene delivery, investigators add growth factors inducing proliferation such as IL-13, PHA in case of human T cells^14^,^41^. Meanwhile, siRNA mediated gene silencing in mouse blood monocytes was not significant compared with other phagocytic cells^7^. Furthermore, CpG ligand mediated siRNA delivery did not reduce the target gene in the human monocyte even with a high uptake^42^, suggesting that monocytes are cells relatively harder to transfect than other phagocytes and have their own endosomal degradation and clearance mechanism. In terms of clinical application, applying targeting moiety for human receptor improves its potency as a drug, jumping the cross-species reactivity problem, but it doesn’t exclude the need for animal studies such as target specificity and efficacy *in vivo*. Humanized mice is an existing animal model for human immune cell study *in vivo*, and genetic engineering technologies have developed mouse strains more suitable for human immune system simulation. A recent paper reported tumor accumulation of human macrophages in a cancer cell grafted to TG mice as the same as in a human patient^43^. However those generations do not reflect whole human inflammatory disease pathology such as disease accumulation of phagocytes^43^,^44^,^45^ in inflammation. Collectively, our data demonstrates the CD64-targeted siRNA delivery as a potent system for human blood monocyte gene delivery.

## Materials and Methods

### Vector construction

An 800 base pair sequence-encoding anti-human CD64 scFv with a C-terminal cysteine residue and a 6x His-tag was synthesized and cloned (Incorporation Bioneer) between xbaI and NotI restriction sites of the pET21a vector (Novagen, Madison, WI) for bacterial expression.

### SiRNAs

Luciferase siRNA conjugated with FITC was synthesized and purchased from ST Pharm. Co. Ltd. SiRNAs for cyclophilin B (siCypB), CD45 (siCD45), and luciferase (siLuci) were purchased from Dharmacon.

### Cell culture

THP-1 and MDA-MB 231 were purchased from KCLB (Korea Cell Line Bank, Korea). THP-1 cells were cultured in RPMI1640 (Welgin, Korea; FBS 10%, penicillin 1%) and MDA-MB 231 cells were cultured in DMEM high glucose (Welgin, Korea; FBS 10%, penicillin 1%). THP-1 cells were passaged to a cell density of 1 × 10^5^ cells/ml.

### Human peripheral blood mononuclear cell

For human peripheral blood cell culture, PBMCs were purchased from StemCell or Lonza and cultured in RPMI1640 (Welgin, Korea; FBS 10%, penicillin 1%).

### Protein expression

For anti-CD64 scFv expression, BL21 (DE3) cells (Novagen, Madison, WI) inoculated with anti-CD64 scFv pET21a vector were cultured in 50 ml of ampicillin containing LB medium at 37 °C. After 4 hours cells were cultured in 500 ml of ampicillin containing LB medium. When O.D_600nm_ reached 0.3–0.5, 1 mM IPTG was added and cells were induced for 16 hours at 26 °C. Induced cells were collected using a centrifuge at 480 g, and pellets were re-suspended in a lysis buffer (pH 8.0) and then sonicated. Soluble protein solutions were collected using a centrifuge at 27,500 g, and the resultant solutions were filtered using a 0.45 μm filter.

### Affinity chromatography purification

Soluble protein solution was loaded onto nickel-agarose resin (Qiagen)-charged column and washed with 10 volume equivalents of washing buffer (25 mM imidazole). Resin bound proteins were eluted with elution buffer (250 mM imidazole) and dialyzed with a dialysis membrane (Spectrum laboratories, CA; MWCO = 12–14,000 Da) using phosphate buffered saline (PBS; pH 7.4).

### SDS-PAGE and western blot

Purified protein was mixed with Laemmli buffer (DTT 5 mM) in a 1:1 volume ratio and incubated for 15 min at room temperature. Samples were loaded onto 12% SDS-PAGE gels and underwent electrophoresis. After electrophoresis, gels were stained with Coomassie Brilliant Blue (Bio-rad) or transferred to a PVDF membrane (Millipore, Billerica, MA) for western blotting. Anti-His tag antibody (1^st^; cell signaling) and anti-rabbit IgG antibody-HRP (2^nd^; Santa Cruz, sc-2004) bound proteins were detected using a Kodak image station.

### Competition assay

THP-1 cells (2 × 10^5^ cells/well) and MDA-MB 231 cells (2 × 10^5^ cells/well) were incubated with purified anti-CD64 scFv (2 μg) in ice cold PBS for 20 min. After washing cells twice with ice cold PBS, cells were stained with anti-human CD64 antibody (FITC) or anti-CCR2 antibody (PE), anti-CD14 antibody (PE) on ice and analyzed with BD FACS Calibur.

### 9-D arginine conjugation

(Npys)C-rrrrrrrrr-NH_2_ peptide was purchased from Peptron (Daejeon, Korea) and resuspended in a 0.1 M potassium phosphate buffer (pH 5.6). Phosphate buffer-dialyzed anti-CD64 scFv (0.5 mg/ml) was mixed with 9-D arginine in a molar ratio of 1:10, incubated for 4 hours in a dark room with gently stirred. After 4 hours of incubation, the solution was dialyzed to remove un-conjugated D-9 arginine (MWCO = 12–14,000 Da).

### Retardation assay

The siLuci (40 pmol) was mixed with anti-CD64 scFv or anti-CD64 scFv-9R in diverse N/P ratios and incubated for 15–20 min at room temperature. Samples were loaded into a 2% agarose gel and electrophoresed, and detection was performed using a Kodak image station.

### Conjugation efficiency

Oligo-arginine conjugation efficiency was quantified with free-thiol quantification kit (M30550, Invitrogen). Simply, free thiol group of scFv, scFv-9R was quantified and relative ratio and conjugation efficiency were calculated.

### Anti-CD64 scFv-9R mediated cell binding of siRNA

For THP-1 binding, anti-CD64 scFv-9R and anti-CD64 scFv were mixed with 75 pmol siLuci (FITC) in an N/P ratio of 4 and incubated for 20 min at room temperature. The 2 × 10^5^ of THP-1 cells were incubated with the anti-CD64 scFv-9R/siLuci (FITC) complex in either the presence or absence of free anti-CD64 scFv protein on ice for 30 min. The cells were washed twice with PBS and analyzed using a BD FACS Calibur.

### Human CD64-specific binding study with CHO-K1 cell line

The hamster ovary cell line CHO-K1 was transfected with human CD64 expression vector (CMV-hCD64 pDNA vector 3 ug with lipofectamine2000) (RC207487, Origene). Seventy-two hours after transfection, 5 × 10^4^ cells/ml were incubated with anti-CD64 scFv-9R/siRNA (FITC) complex (40 pmol, NP ratio 4) on ice for 30 min and analyzed with flowcytometry.

### Anti-CD64 scFv-9R mediated siRNA delivery (THP-1, MDA-MB 231)

The siLuci (150 pmol) conjugated with FITC was incubated with anti-CD64 scFv-9R in diverse N/P ratios for 20 min. The anti-CD64 scFv-9R/siLuci (FITC) complex prepared in serum-free media was mixed with 2 × 10^5^ pre-seeded THP-1 and MDA-MB 231 cells. After 4 hours of incubation, media was replaced with serum-containing media (FBS 10%, penicillin 1%) and incubated for an additional 8–10 hours for flowcytometry analysis or 24 hours for total RNA isolation. For RT-PCR, cyclophilin B mRNA was normalized with beta-actin mRNA. Transfection with Lipofectamine2000 was performed according to manufacturer’s instructions.

### Anti-CD64 scFv-9R mediated siRNA delivery with human peripheral blood mononuclear cell (PBMC)

After thawing and passaging, the 1–2 × 10^6^ PBMCs were seeded in 24-well plates with RPMI1640 media (FBS 10%, penicillin 1%). After 22–24 hours, 110 pmol to 150 pmol of siLuci (FITC) complexed with anti-CD64 scFv-9R prepared in serum free media were mixed with PBMCs. After 4 hours of incubation, media was replaced with serum- containing media and incubated for an additional 7–9 hours. The PBMCs were stained with anti-CD14 antibody (PE) or anti-CD3 antibody (PE), as well as anti-CD19 antibody (PE) for flowcytometry analysis. For RT-PCR, fluorescence associated cell sorting (FACS) sorted 2 × 10^5^ CD14 (+) cells and lymphocytes were seeded in 24 well plate and treated with anti-CD64 scFv-9R/siCypB complex (110 pmol, N/P ratio = 4). After 4 hours of incubation, media was replaced with serum-containing media and incubated for an additional 20–22 hours for total RNA isolation. For western blot, 2 × 10^6^ PBMCs/well (24 well plate) were seeded and treated with anti-CD64 scFv-9R/siRNA complex. 30 hours after treat, cells were stained anti-CD14 antibody (PE) and sorted with FACS Aria and sorted cells were used for western blot sampling. For the surface CD45 silencing test, total PBMCs were co-stained with anti-CD45 antibody (PerCP Cy5.5), anti-CD14 antibody (FITC), and monocytes, lymphocytes were gated and analyzed for surface CD45 measurement.

### Annexin V assay

Pre-seeded PBMCs (2 × 10^6^cells/well, 24 well plate) were treated with either the siLuci or anti-CD64 scFv-9R/siLuci complex (150 pmol, N/P ratio = 4). After 24 or 48 housr of incubation, PBMCs were stained with Annexin V (PE) and total live cells were gated for analysis using flowcytometry. As a positive control, PBMCs were heat shocked (50 °C, 30 min) and stained with Annexin V (PE).

### MTT assay

The FACS sorted 2 × 10^5^ of CD14 (+) monocytes and lymphocytes were seeded in 24 well plate and treated with anti-CD64 scFv-9R/siRNA complex (siRNA 150 pmol, N/P ratio 4). MTT solution was added after 24 hours and colorimetric analysis was performed.

### Immune response assay

Pre-seeded PBMCs (2 × 10^6^cells/well) were treated with naked siLuci (150 pmol) or siLuci complexed with anti-CD64 scFv-9R (N/P ratio = 4 and 6). After 24 hours total RNA was isolated and the mRNA levels were measured and normalized by beta-actin mRNA.

### *In vivo* human monocyte targeting effect

The 5–7 weeks aged NOD-scid (Jackson laboratory) mice were engrafted with FACS sorted human CD14 (+) monocytes (5–7.5 × 10^5^ cells per mouse) by intravenous injection. After 1–2 hours, anti-CD64 scFv-9R/siRNA complexes (siRNA (FITC) 500 pmol, N/P ratio 4) were injected intravenously and incubated for additional 1–2 hours in dark. Total blood was harvested from mouse heart and lysed with red blood cell lysis buffer (Sigma Aldrich) and stained with mouse, human CD45 antibody (PerCP-Cy5.5) for flowcytometry analysis. The blood monocytes were indicated with forward scattering (FSC) and CD45 staining. All animal experimental procedures with NOD-scid mice were reviewed and approved by the Institutional Animal Care and Use Committee (IACUC) of the Hanyang University (HY-IACUC-16–0126) and was performed in accordance with the relevant guidelines. The mice were housed under specific pathogen free conditions.

### Blood profile

The NOD-scid (Jackson laboratory) mice were engrafted with FACS sorted human CD14 (+) monocytes (5–7.5 × 10^5^ cells per mouse) and injected with scFv-9R/siRNA complex (siRNA (Cy5.5) 500 pmol, N/P ratio 4). At each time points, 30–50 ul of blood was harvested from orbital vein and incubated 30 min for serum isolation. Serum was 3 times diluted in PBS and detected for Cy5.5 (excitation: 695 nm, emission: 720 nm).

### Statistical analysis

Data are presented as the mean ± standard deviations. Statistical analyses were performed using the Student’s t-test, One-way ANOVA and Tukey posttest and the GraphPad Prism 5 Project software.

### Flowcytometry analysis

All treated replicates were assembled and prepared in one tube and performed with BD FACS Calibur (BD biosciences, San Diego, CA) and analyzed with CellQuest Software (BD Pharmingen, San Jose, CA).

## Additional Information

**How to cite this article**: Yong, S.-B. *et al*. Human CD64-targeted non-viral siRNA delivery system for blood monocyte gene modulation. *Sci. Rep.* **7**, 42171; doi: 10.1038/srep42171 (2017).

**Publisher's note:** Springer Nature remains neutral with regard to jurisdictional claims in published maps and institutional affiliations.

## Supplementary Material

Supplementary Information
