# Supplementary material for: Human CD64-targeted non-viral siRNA delivery system for blood monocyte gene modulation
Source: Sci Rep. 2017 Feb 7;7:42171. doi: 10.1038/srep42171 (PMC5294565; doi:10.1038/srep42171)
Supplement: Supplementary Information [file srep42171-s1.pdf]

Supplementary information

Human CD64-targeted non-viral siRNA delivery system for blood monocyte gene modulation

Seok-Beom Yong, Hyung Jin Kim, Jang Kyoung Kim, Jee Young Chung, Yong-Hee Kim*

Department of Bioengineering, Institute for Bioengineering and Biopharmaceutical Research,

BK 21 Plus Future Biopharmaceutical Human Resources Training and Research Team,

Hanyang University, 133-791 Seoul, Republic of Korea

***Correspondence: Yong-Hee Kim, Department of Bioengineering, Hanyang University, 17,**

Haendang-dong, Seongdong-gu, Seoul, 133-791, Republic of Korea. E-mail:

yongheekim@hanyang.ac.kr

Supplementary data

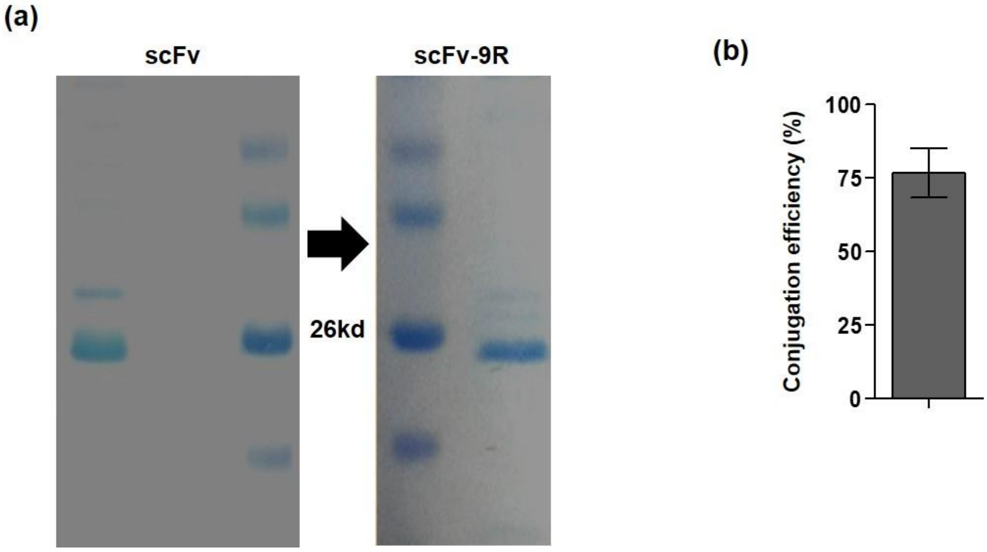

Figure S1. SDS-PAGE of scFv-9R. After oligo-arginine conjugation and dialysis, scFv-9R was confirmed in SDS-PAGE and compared with scFv. The scFv and scFv-9R did not show significant difference in electrophoresis-mobility. (fig. a) With free thiol quantification assay, relative free thiol ratio was calculated and conjugation efficiency was ~77%. (fig. b)

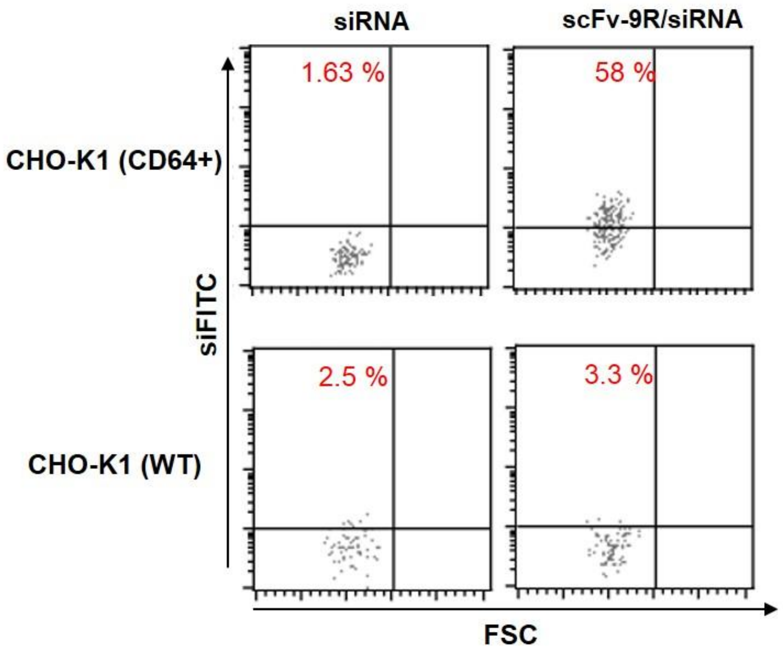

Figure S2. Human CD64-mediated cell binding of scFv-9R/siRNA complex with CHO-K1 cell. Human CD64-CMV plasmid (MC207487, Origene) was transfected with hamster ovary cell CHO-K1 and cell binding was confirmed. Transfected CHO-K1 showed increased scFv-9R/siRNA contrast to wild type CHO-K1 cell. (siRNA (FITC) 40pmol, N/P ratio 4)

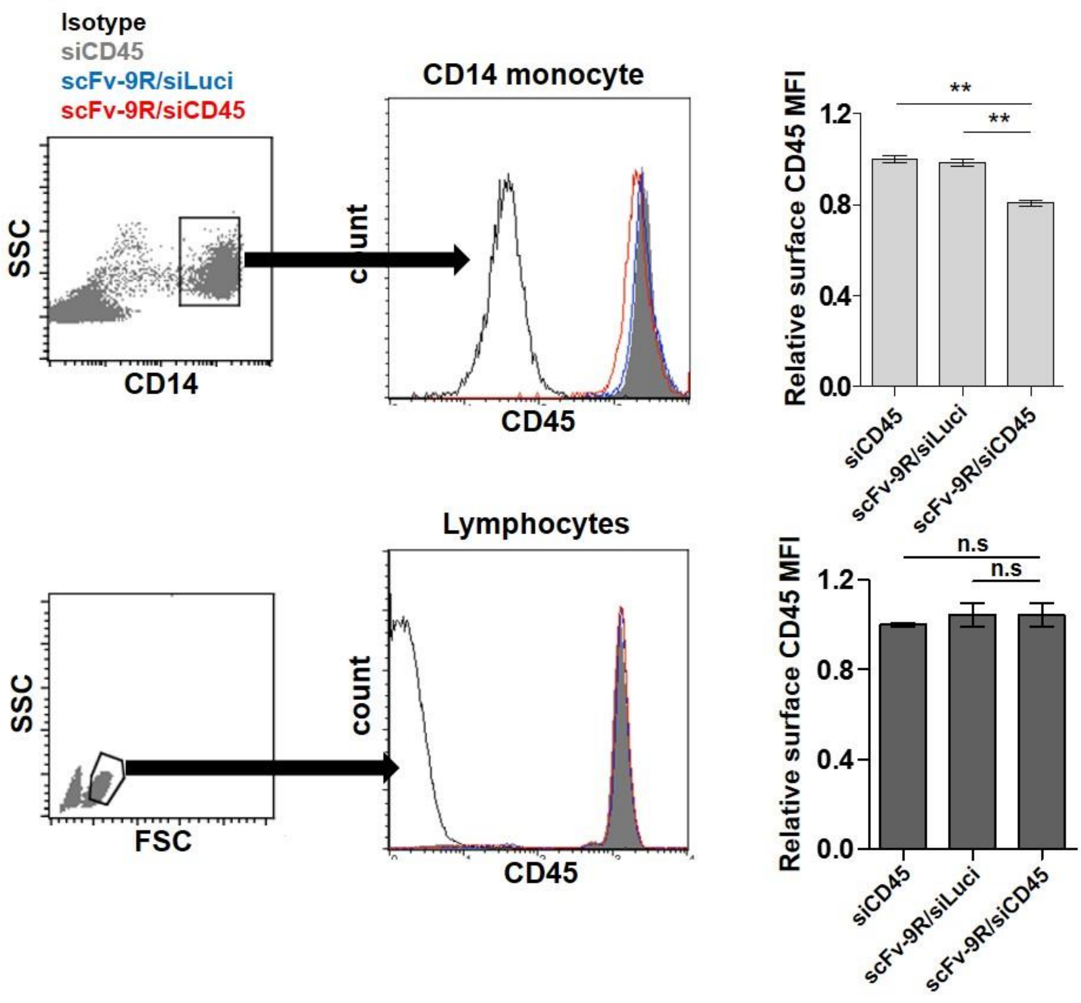

Figure S3. Flowcytometric analysis of surface CD45 silencing in PBMC. Total PBMCs were stained with anti-CD14 antibody (FITC), anti-CD45 antibody (PerCP-Cy5.5). The CD14 gated cells (monocytes) and side scattering gated cells (lymphocytes) were analyzed for surface CD45 expression

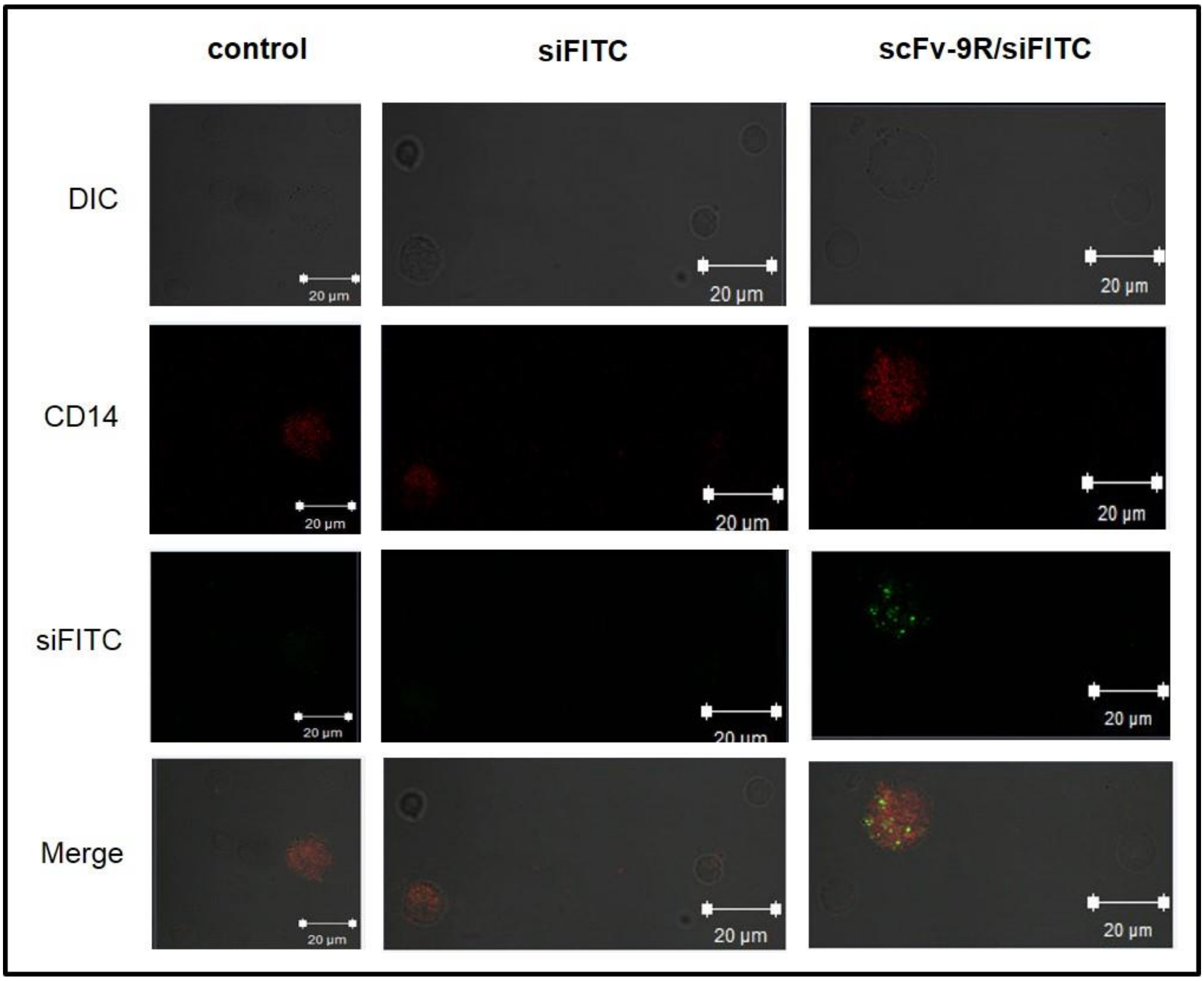

Figure S4. Confocal microscopy image of PBMCs. The scFv-9R/siFITC (N/P ratio 5, 150pmol) treated PBMCs were stained with anti-CD14 antibody (PE) and analyzed with confocal microscopy.

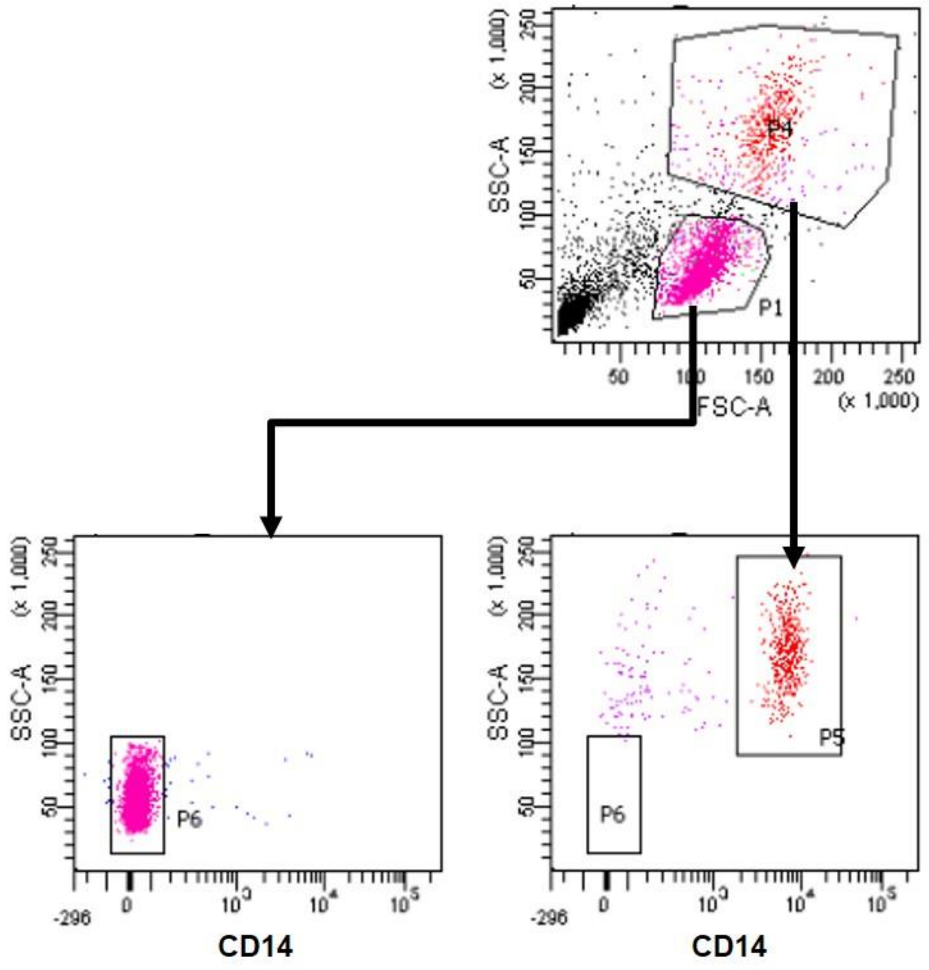

Figure S5. FACS sorting data. Total PBMCs (1.5~3x10^7 cells) were stained with anti-CD14 antibody (PE) and CD14 (+) Monocytes and lymphocytes were sorted from peripheral blood mononuclear cells (PBMCs).
